# Supplementary material for: Dynamics of accessible chromatin regions and subgenome dominance in octoploid strawberry
Source: Nat Commun. 2024 Mar 20;15:2491. doi: 10.1038/s41467-024-46861-0 (PMC10954716; doi:10.1038/s41467-024-46861-0)
Supplement: Supplementary file 1 — Supplementary Information [file 41467_2024_46861_MOESM1_ESM.pdf]

Dynamics of accessible chromatin regions and subgenome dominance in octoploid strawberry

Fang *et al*.

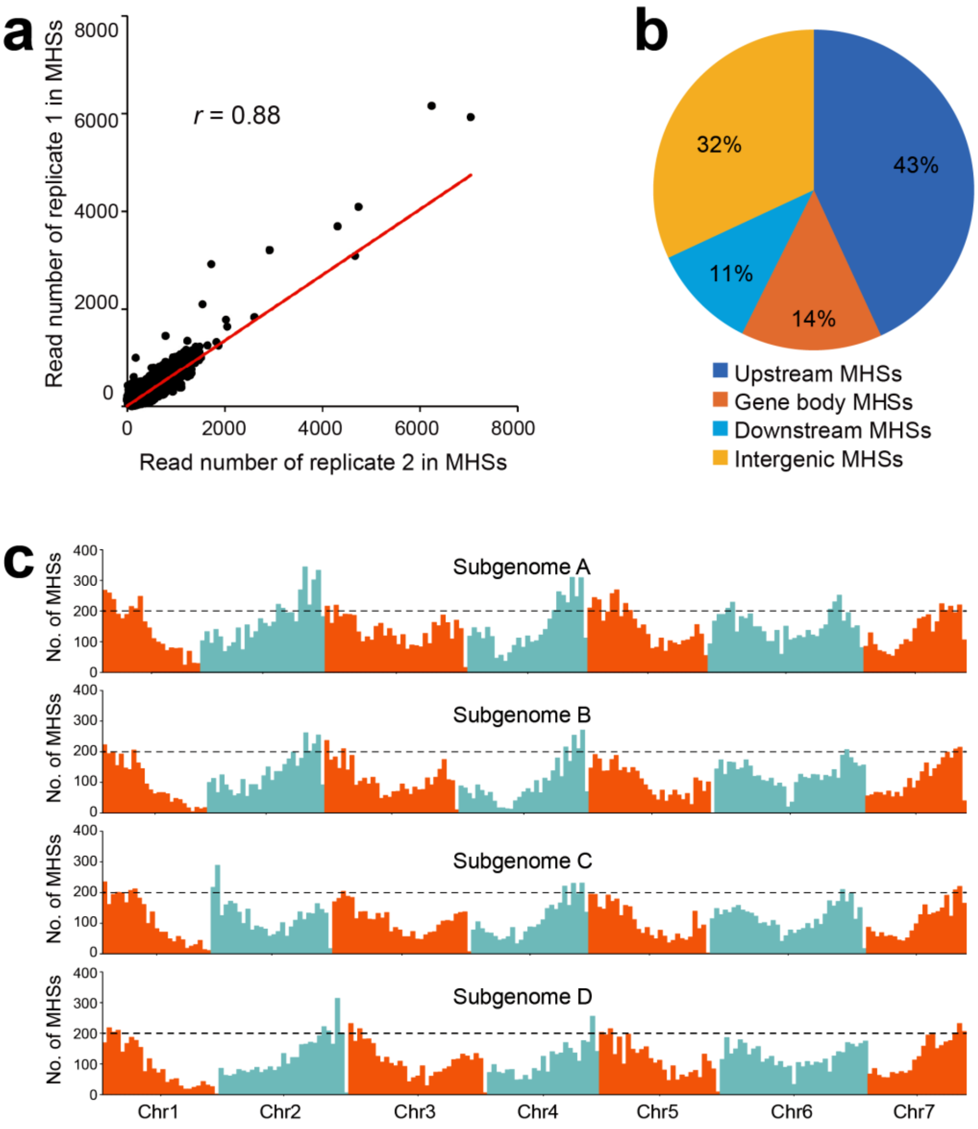

Supplementary Figure 1. MHSs and their distribution in strawberry genome. (**a**) Correlation of MH-seq data between two biological replicates derived from strawberry leaf tissue. The number of MH-seq reads in each MHS was counted and used to detect the correlation between the two replicates. The Pearson correlation coefficients (*r*) are shown in the figure. (**b**) Genomic locations of MHSs. (**c**) Distribution of MHSs across the 7 chromosomes from each of the four subgenomes. The *y* axes show the numbers of MHSs in 1-Mb windows.

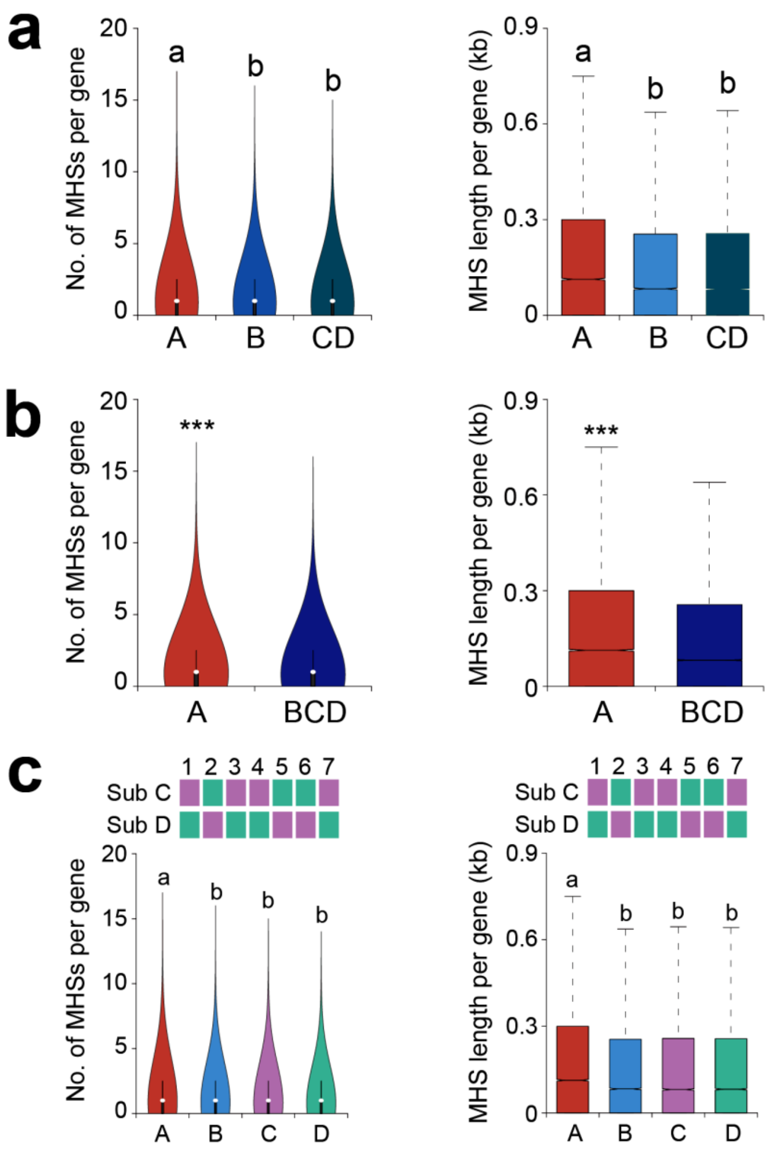

Supplementary Figure 2. Comparison of chromatin accessibility between subgenome A and combined subgenomes B/C/D. (a) The total number of MHSs (left) and MHS length (right) were combined from subgenomes C and D. The combined values were normalized by dividing to the total number of genes from subgenomes C/D. **(b)** The total number of MHSs (left) and MHS length (right) were combined from subgenomes B/C/D. The combined values were normalized by dividing to the total number of genes from subgenomes B, C, and D. (**c**) Average number of MHSs per gene (left) and average MHS length per gene (right) in subgenomes A, B, and newly created C, D. Purple boxes indicate the chromosomes from subgenome C and green boxes indicate chromosomes from subgenome D. We exchanged the chromosome 2, 5, and 6 between subgenome C and D. The total number of MHSs and the total MHS lengths were normalized in the four subgenomes by dividing to their total number of genes from each subgenome. The lower and upper boundaries of each box indicate 25th and 75th percentile, the center line indicates the median, and the whiskers extend to 1.5× IQR in (a-c). Means that do not share a letter are significantly different ($p<0.01$, one-way ANOVA with Games-Howell *post-hoc* test). ***$p<0.001$, Mann-Whitney *U* test. Source data are provided as a Source Data file.

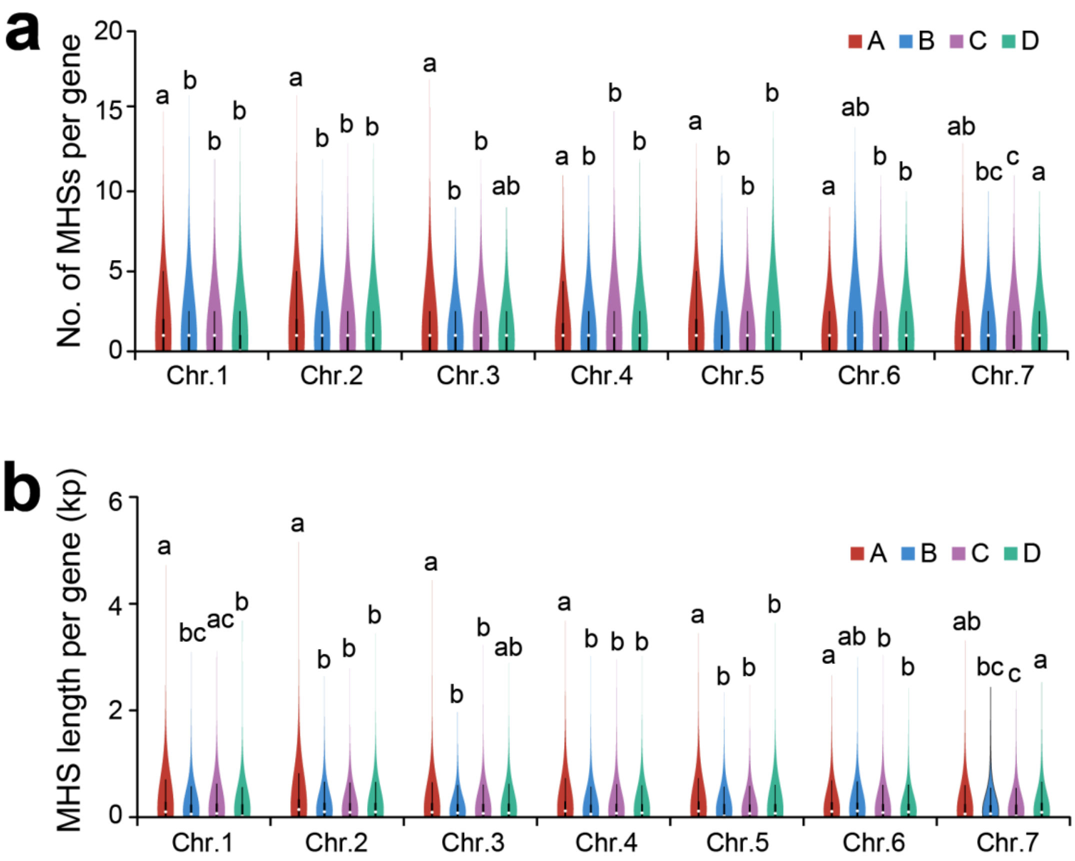

Supplementary Figure 3. Comparisons of chromatin accessibility among the four chromosomes in each homoeologous group. (a) Average number of MHSs per gene of every chromosome in each homoeologous group. **(b)** Average MHS length per gene of every chromosome in each homoeologous group. The total number of MHSs and MHS length were normalized by dividing to their total number of genes from each chromosome. Means that do not share a letter are significantly different ($p<0.01$, one-way ANOVA with Games-Howell *post-hoc* test). Source data are provided as a Source Data file.

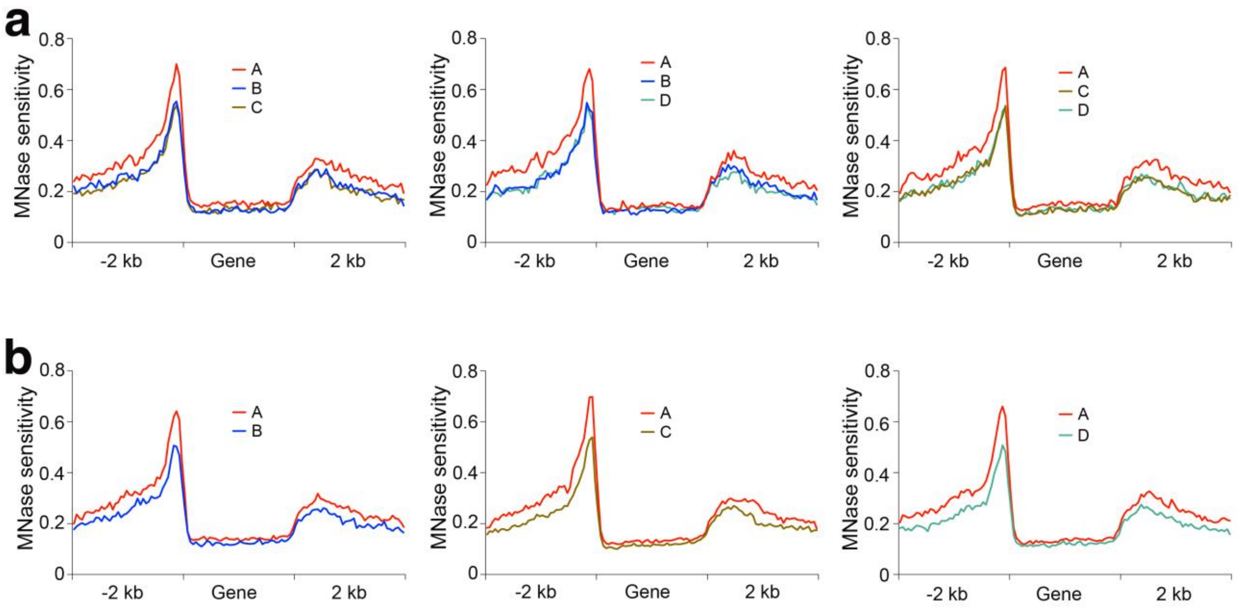

Supplementary Figure 4. Correlation of the levels of gene expression and chromatin accessibility associated with subgenome A. (**a**) MH-seq profiles of the highly expressed genes from subgenome A and their homoeologs with lower expression levels in only two of the three submissive subgenomes (n (left) = 707; n (middle) = 646; n (right) = 705). (**b**) MH-seq profiles of the highly expressed genes from subgenome A and their homoeologs with lower expression levels in only one of the three submissive subgenomes (n (left) = 1,179; n (middle) = 1,342; n (right) = 1,467).

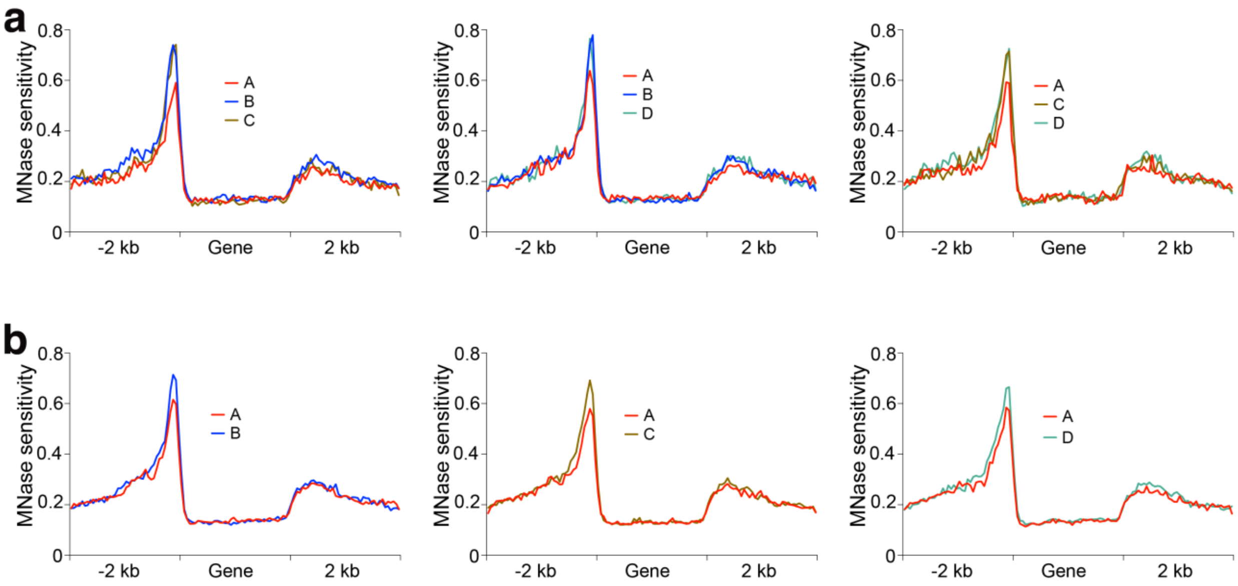

Supplementary Figure 5. Chromatin accessibility associated with the lowly expressed genes from subgenome A. (**a**) MH-seq profiles of the lowly expressed genes from subgenome A and their homoeologs with higher expression levels in only two of the three submissive subgenomes (n (left) = 394; n (middle) = 371; n (right) = 299). (**b**) MH-seq profiles of the lowly expressed genes from subgenome A and their homoeologs with higher expression levels in only one of the three submissive subgenomes (n (left) = 1,046; n (middle) = 949; n (right) = 931).

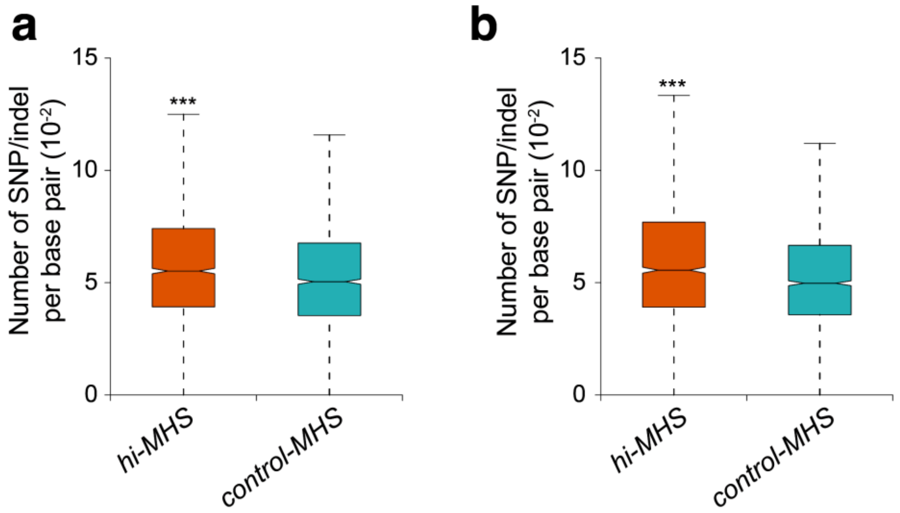

Supplementary Figure 6. Sequence variation between hi-MHSs and their homoeologs. (**a**) The number of SNPs/INDELs between hi-MHSs from subgenome A and their homoeologs from subgenome C. The numbers of SNPs/INDELs between control-MHS from subgenome A and their homoeologs from subgenome C were calculated and used as a control. The number of SNP/INDEL was normalized by dividing to the alignment length between MHS and its homoeolog. (**b**) The number of SNPs/INDELs between hi-MHSs from subgenome A and their homoeologs from subgenome D. The lower and upper boundaries of each box indicate 25th and 75th percentile, the center line indicates the median, and the whiskers extend to 1.5× IQR in (a) and (b). ***p<0.001, Mann-Whitney U test. Source data are provided as a Source Data file.

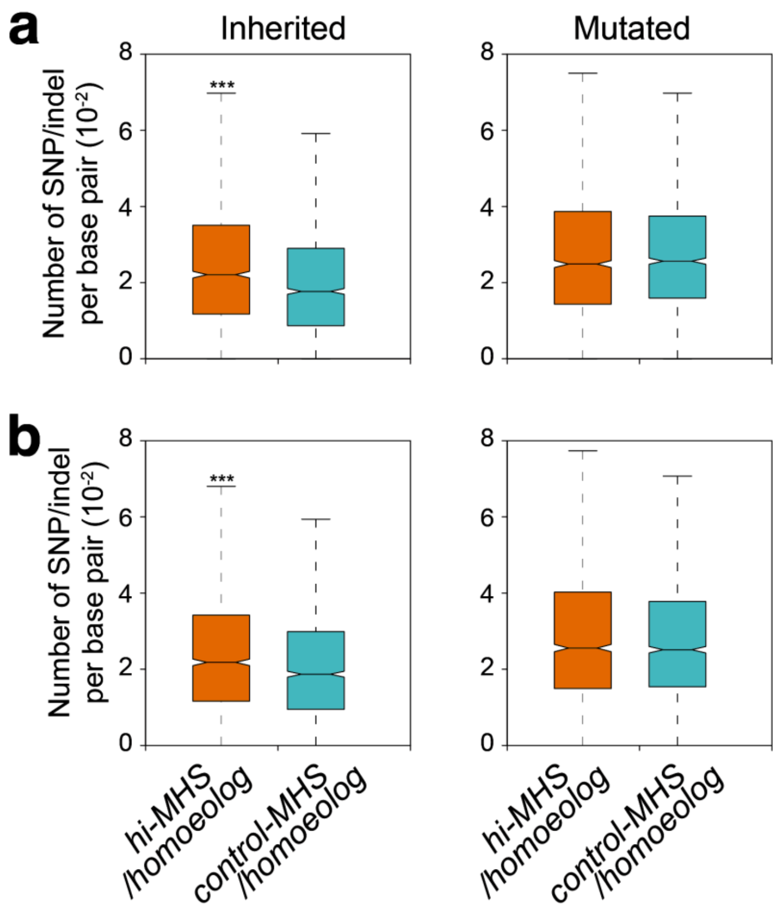

Supplementary Figure 7. Sequence variation and MHS divergence between subgenome A and subgenomes C/D. (a) The number of inherited (left panel) and mutated (right panel) types of SNP/INDEL in hi-MHS (A)/homoeolog (C) pairs and control-MHS (A)/homoeolog (C) pairs. **(b)** A similar comparison between subgenome A and subgenome D. The number of SNP/indel was normalized by dividing to the overlapped length between MHS and its homoeolog. The lower and upper boundaries of each box indicate 25th and 75th percentile, the center line indicates the median, and the whiskers extend to 1.5× IQR in (a) and (b). ***p<0.001, Mann-Whitney U test. Source data are provided as a Source Data file.

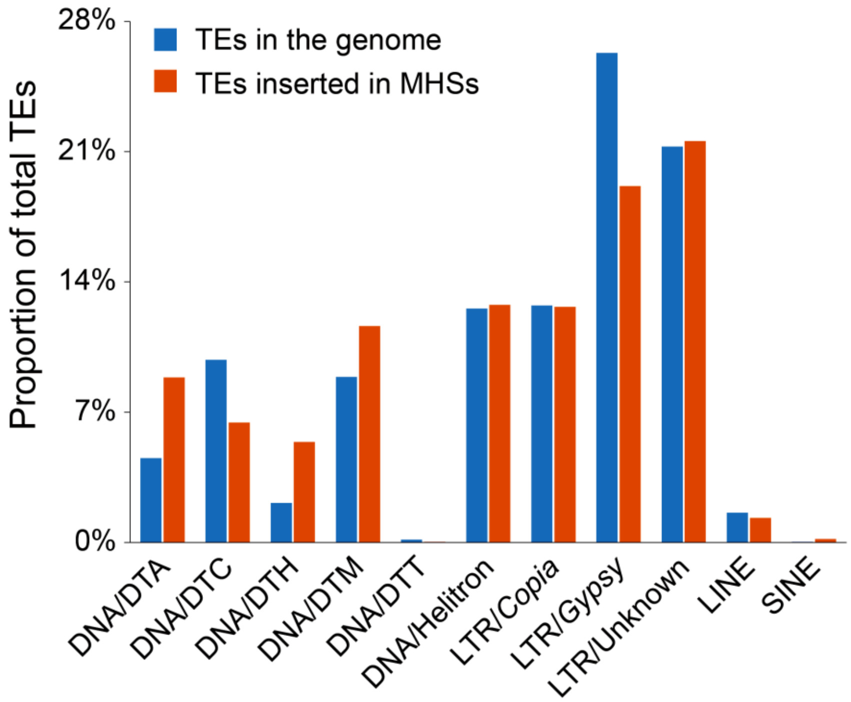

Supplementary Figure 8. Classes of TEs that were identified between two fragmoeologs. The percentage of each class of TE within fragmoeologs is compared to the percentage of the same class of TE in the strawberry genome.

Supplementary Table 1. Number of genes and MHSs identified in each subgenome.

Subgenome	Genes	MHSs	Singleton MHSs	Syntenic MHSs
A	27,398 (7,602)*	28,798	3,649	25,149
B	25,461 (6,764)	23,237	1,827	21,410
C	24,505 (6,526)	22,436	1,662	20,774
D	23,911 (6,230)	22,370	1,622	20,748

* Number in the parentheses indicates genes specific to the subgenome
